# MAR1 suppresses inflammatory response in LPS-induced RAW 264.7 macrophages and human primary peripheral blood mononuclear cells via the SIRT1/PGC-1α/PPAR-γ pathway

**DOI:** 10.1186/s12950-021-00271-x

**Published:** 2021-02-08

**Authors:** Wei Wang, Rong-Li Xu, Ping He, Rui Chen

**Affiliations:** 1 Department of Emergency, Hainan General Hospital, Hainan Affiliated Hospital of Hainan Medical University, No.19, Xiuhua Road, 570311 Haikou, Hainan Province People’s Republic of China; 2 Department of Cardiology, Hainan General Hospital, Hainan Affiliated Hospital of Hainan Medical University, No.19, Xiuhua Road, 570311 Haikou, Hainan Province People’s Republic of China; 3 Department of Medical Intensive Care Unit, General Hospital of Southern Theater Command, PLA, No.111, Liuhua Road, 510010 Guangzhou, People’s Republic of China

**Keywords:** MAR1, SIRT1, PGC-1α, PPAR-γ, Sepsis

## Abstract

**Background:**

Sepsis is a complex syndrome characterized by a dysregulated inflammatory response to systemic infection and leads to shock, multiple organ failure and death especially if not recognized early and treated promptly. Previous studies have suggested Maresin 1 (MAR1) can alleviate systemic inflammation in sepsis, but its mechanism has not been clarified.

**Methods:**

RAW 264.7 cells and human primary peripheral blood mononuclear cells (hPBMCs) were pretreated with LPS and MAR1. The mRNA expression and supernatant levels of pro-inflammatory cytokines, tumor necrosis factor (TNF-α), interleukin (IL)-1β and IL-6 were evaluated by RT-qPCR and ELISA, respectively. The expression levels of Sirtuin 1 (SIRT1), peroxisome proliferator-activated receptor γ coactivator-1α (PGC-1α), and Peroxisome proliferator-activated receptor gamma (PPAR-γ) were determined by RT-qPCR and Western blot analysis, respectively.

**Results:**

Our results show that LPS-induced inflammation increased the expression and secretion of proinflammatory cytokines TNF-α, IL-1β and IL-6 and induced suppression of SIRT1, PGC-1α, and PPAR-γ expression, which could be reversed by MAR1. And the effect of MAR1 was eliminated by repression of SIRT1/PPAR-γ and enhanced by PGC-1α overexpression.

**Conclusions:**

MAR1 suppressed inflammatory response in LPS-induced RAW 264.7 macrophages and hPBMCs via the SIRT1/PGC-1α/PPAR-γ pathway.

## Introduction

Sepsis, defined as a syndrome of systemic inflammatory response (SIRS) caused by infection as a result of an infective process, is a rapidly progressive and life-threatening syndrome. Severe sepsis with multiple organ dysfunctions is still the main cause of death in intensive care unit patients [1, 2]. During sepsis, inflammation response can be activated in both macrophages and monocytes, and the inflammatory factors released by macrophages may accelerate the sepsis-induced cell and tissue injury [3, 4]. Lipopolysaccharide (LPS), a component of the outer membrane of Gram-negative bacteria, interacts with specific receptors on host effector cells and induces the release of proinflammatory cytokines [5]. Excessive production of these cytokines can lead to an uncontrolled inflammatory response [6]. Therefore, LPS is widely held to be a mediator of the inflammatory events associated with sepsis [7]. LPS-treated macrophages were adopted as an *in vitro* model of endotoxin-induced inflammation during sepsis [8], such as using the murine macrophage cell line RAW264.7 [9, 10].

Specialized pro-resolving mediators such as resolvins, protectins, and maresins, actively turn off the inflammatory response by acting on different G protein-coupled receptors expressed on immune cells that activate the dual anti-inflammatory process [11]. Maresin 1 (MAR1) from polyunsaturated fatty acids is one of the most recently identified members of the family of anti-inflammatory lipid mediators and has shown anti-inflammatory and proresolving activity in zymosan-induced peritonitis [12]. Many studies have shown that MAR1 shows proresolving and anti-inflammatory effects in a variety of diseases [12–14]. However, the specific mechanism of its role in sepsis remains unclear. In previous studies, MAR1 has been shown to reduce proinflammatory response and mitochondrial damage by activating SIRT1 signaling to protect mouse brain tissue and neurons from ischemia/reperfusion injury [15, 16]. A large number of studies have shown that sirtuin 1 (SIRT1) is a highly conserved mammalian NAD(+)-dependent protein deacetylase and is involved in multiple biological processes [17]. Therefore, it is not surprising that the changes in SIRT1 activity or expression profile are strongly correlated with metabolic processes, oxidative stress, and the pathogenesis of inflammation [17, 18]. Furthermore, SIRT1 knockout mice have increased sensitivity to septic-induced inflammatory lung injury [19, 20]. Meanwhile, SIRT1 suppresses acute lung inflammation during sepsis by controlling the activation of inflammatory pathway [21], so it is highly possible that MAR1 reduce the inflammatory response by activating SIRT1 in sepsis. Peroxisome proliferator-activated receptor-γ co-activator-1α (PGC-1α) is one of the three known co-activators in the PGC-1 family [22], it can bind to multiple transcription factors, effectively promote mitochondrial biogenesis and reduce mitochondrial damage [23]. PGC-1α can not only be activated by SIRT1 through deacetylation, but also act as a transcription coactivator to enhance the activity of peroxisome proliferator-activated receptor γ (PPARγ) [24, 25]. PPARγ is a member of the nuclear receptor superfamily of ligand-induced transcription factors, which mainly controls gene expression involved in adipogenesis, lipid metabolism, and inflammation [26]. Likewise, PPAR-γ activation is an effective intervention to prevent or restore septic myocardial dysfunction and has proven to be a promising treatment strategy for sepsis [27]. From this result, we initially proposed that MAR1 reduces the inflammation response in sepsis may be related to the activation of SIRT1/PGC-1α/PPAR-γ axis.

In our current study, the relationship between reduction of inflammatory response by MAR1 and SIRT1/PGC-1α/PPAR-γ signaling axis was systematically investigated and intended to explain the specific mechanism of MAR1 in *in vitro* sepsis models of LPS-induced RAW 264.7 cells and human primary peripheral blood mononuclear cells (hPBMCs).

## Materials and methods

### Cell culture and treatment

The mouse mononuclear macrophage leukemia cell line RAW264.7 was purchased from the American Type Culture Collection (ATCC), and primary human peripheral blood mononuclear cells (hPBMCs) was purchased from PriCells (Wuhan, China). Cells were cultured in DMEM containing 10 % fetal bovine serum (FBS, Gibco; Thermo Fisher Scientific) and 1 % penicillin (100 U/mL)/streptomycin (100 µg/mL) (Hyclone; GE Healthcare Life Sciences) in a 37℃ in an incubator containing 5 % CO_2_ at 37 °C. For establishment of *in vitro* model, lipopolysaccharide (LPS, 10 µg/ mL, Sigma-Aldrich, St. Louis, MO, USA) was used to treat the cells for 24 h. For treatment of MAR1, cells were treated with different concentrations (0.1 nM, 1 nM, 10 nM and 100 nM) of MAR1 (cat. No. HY-116,429; MedChemExpress, NJ, USA) for 12 h before LPS treatment. For analysis of time effects, cells were treated with 10 nM MAR1 with different time duration (0 h, 4 h, 6 h, 8 h and 12 h).

### Real‐time qPCR analysis

Total RNA was extracted from cells using TRIzol reagent (Invitrogen). After total RNA was reverse transcribed into cDNA by using a Prime-Script™ One Step RT-qPCR kit (Takara Biotechnology Co., Ltd., Dalian, China), PCR reactions for SIRT1, PGC-1α, PPAR-γ, IL-1β, IL-6 and TNF-α mRNA were performed on a LightCycler 480 (Roche, Mannheim, Germany) system with GAPDH used as an internal control. The light cycler DNA master SYBR green I kit (Roche Molecular Biochemicals, Mannheim, Germany) was then used for PCR experiments. The following primers were used: SIRT1 Forward: 5′-GGTGTTAAATACCAAACTGC-3′ and reverse: 5′-AGGAGTGATGTTCAAAATG-3′; PGC-1α Forward: 5’-AATTCACAATCACAGGATCAGAACA3’ and reverse: 5’-ACTTAAGGTGCGTTCAATAGTCTT-3’; PPAR-γ Forward: 5’-TTGGCCATATTTATAGCTGTCATTATT-3′ and reverse: 5’- TGTCCTCGATGGGCTTCA-3’; TNF-α Forward: 5’-GAGCTGTGGGGAGAACAAAAGGA-3′ and reverse: 5’- TTGGCCCTTGAAGAGGACCTG-3’; IL-1β Forward: 5’-GAC CTT CCA GGA TGA GGA CA-3′ and reverse: 5’-AGC TCATATGGGTCCGACAG-3’; IL-6 Forward: 5’-TCC AGT TGC CTTCTT GGG AC-3′ and reverse: 5’-GTGTAATTAAGCCTCCGACTTG-3’; GAPDH Forward: 5’-AGAAGGCTGGGGCTCATTTG-3’ and reverse: 5’-AGGGGCCATCCACAGTCTTC-3. The relative expression of the target genes was calculated using the 2^−ΔΔCt^ method. All experiments were repeated in triplicate.

### ELISA assay

The contents of IL-1β, IL-6 and TNF-α in the cell supernatant were determined using an ELISA assay. Centrifuge at 4000 r/min for 15 min at 4 °C to collect the supernatant. ELISA kits were obtained from Boster (Boster Biological Technology, Wuhan, China), and each sample was measured three times according to the manufacturer’s instructions.

### Western blot analysis

Cells were washed in phosphate-buffered saline (PBS), then in 10 mM TrisHCl (pH7.5), 100 mM NaCl, 1 % NP-40, 50 mM NaF, 2 mMEDTA (pH8.0), 1 mM PMSF, Cells were lysed in 10 µg/mL leupeptin, and 10 µg/mL aprotinin. The mixture was collected as a whole cell extract. The protein extract was separated by sodium lauryl sulfate polyacrylamide gel electrophoresis (SDS-PAGE) and transferred to a polyvinylidene fluoride (PVDF) membrane (immunoblotted PVDF membrane, Bio-Rad). The membrane was blocked with Tris buffered saline and 3 % bovine serum albumin (BSA) in Tween 20 (TBST, pH 7.4) for 1.5 h, and then specific primary antibodies with SIRT1, PGC-1α and PPAR-γ treated at 4 °C overnight. GAPDH was used as a protein-loading control. All antibodies were purchased from Proteintech (ProteinTech Group, Chicago, IL, USA.) The membrane was then treated with horseradish peroxidase (HRP) -conjugated secondary antibody (1: 1,000) for 2 h. The bands were visualized using an enhanced chemiluminescence system (ECL, Thermo Fisher Scientific) and LAS image software (Fuji, New York, New York, USA).

### Cell transfection

GenePharma (Shanghai, China) synthesized PGC-1α overexpression, sh-Sirt1 vector, sh-PPAR-γ vector and corresponding negative controls. Cells (3 × 10^5^) were seeded into 6-well plates for cell transfection after cell confluence of 40–60 %. Replace with new medium and prepare cell transfection buffer. Incubate 5 µL/Wells of lipofectamine 2000 (Thermo, USA) and 250 µL of Opti-MEM medium (Gibco, USA) for 5 min at room temperature and mix with PGC-1α, sh-Sirt1 vector, sh-PPAR-γ vector and negative control (5 µL/well) 250 µL of Opti-MEM medium for 20 min. Then, inoculate the cells into a 6-well plate and incubate in the incubator for 6 h. Then, the cells are incubated in 10 % FBS continuous culture in new DMEM medium (Gibco, USA).

### Statistical analysis

Each experiment was repeated at least 3 times. Data were analyzed using GraphPad Prism 6 (GraphPad software, San Diego, California, USA). The data were expressed as mean ± SD. with either unpaired two-tailed t-test or one-way ANOVA as appropriate. *P* < 0.05 was considered statistically significant.

## Results

### MAR1 inhibited the inflammatory response of RAW264.7 cells and hPBMCs induced by LPS in a dose-dependent manner

**Fig. 1:**
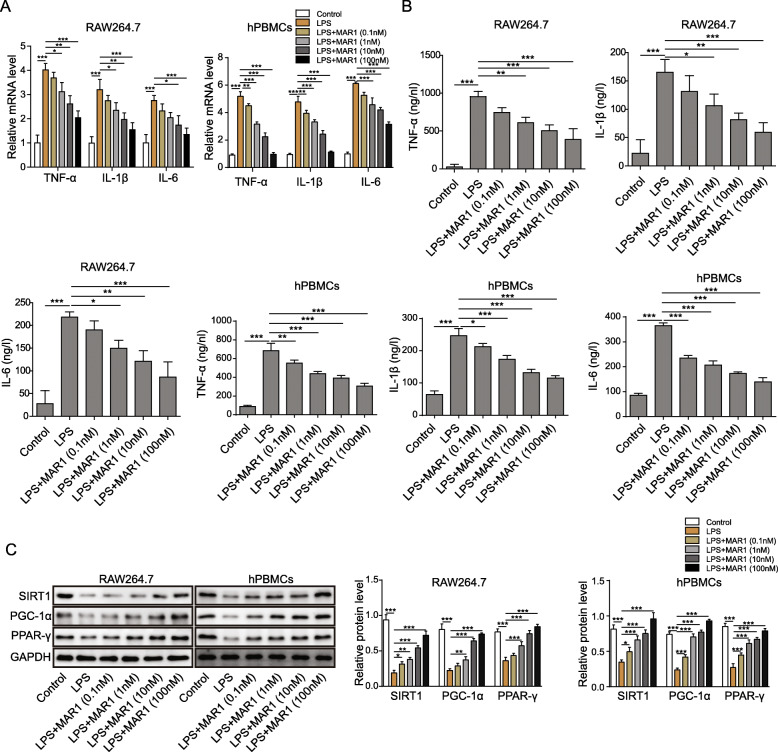
MAR1 inhibited the inflammatory response of RAW264.7 cells and hPBMCs induced by LPS in a dose-dependent manner. RAW264.7 cells and hPBMCs were pretreated with different concentrations (0.1 nM, 1 nM, 10 nM and 100 nM) of MAR1 for 12 h and then exposed to LPS (10 µg/mL) for 12 h. **a**, TNF-α, IL-1β and IL-6 expression were detected by RT-qPCR; **b**, TNF-α, IL-1β and IL-6 secretion were measured using ELISA. **c**, The protein levels of SIRT1, PGC-1α and PPAR-γ were evaluated by Western blot. Data were shown as the means ± SD. Each experiment was repeated in triplicate. *p < 0.05, **p < 0.01, ***p < 0.001

LPS endotoxins are widely used as experimental models of systemic bacterial infection and trigger inflammatory factors such as TNF-α, IL-1β and IL-6 [28]. The changes of inflammatory factors in LPS-treated RAW264.7 cells and hPBMCs under the intervention of different concentrations of MAR1 were analyzed by RT-qPCR. And we found that MAR1 inhibited the LPS-induced increase of the mRNA levels of proinflammatory factors in a dose-dependent manner in both RAW264.7 cells and hPBMCs (Fig. 1a). Next, we tested the secretion of proinflammatory factors TNF-α, IL-1β, and IL-6 in LPS-induced RAW264.7 cells and hPBMCs and the effect of MAR1 on their secretion. The results showed that LPS increased secretion of TNF-α, IL-6 and IL-1β in both RAW264.7 cells and hPBMCs, which can be reversed by MAR1 in a dose-dependent manner (Fig. 1b). In addition, we also performed Western blot assay to evaluate the levels of SIRT1, PGC-1α and PPAR-γ protein, and the results showed that the expression of SIRT1, PGC-1α and PPAR-γ protein were significantly down-regulated in both RAW264.7 cells and hPBMCs caused by LPS (*p* < 0.001) (Fig. 1c) and upregulated by MAR1 in a dose-dependent manner. These results indicate that MAR1 reduces the inflammatory response of RAW264.7 cells and hPBMCs induced by LPS in a dose-dependent manner.

### MAR1 inhibited the inflammatory response of RAW264.7 cells and hPBMCs induced by LPS in a time-dependent manner

**Fig. 2:**
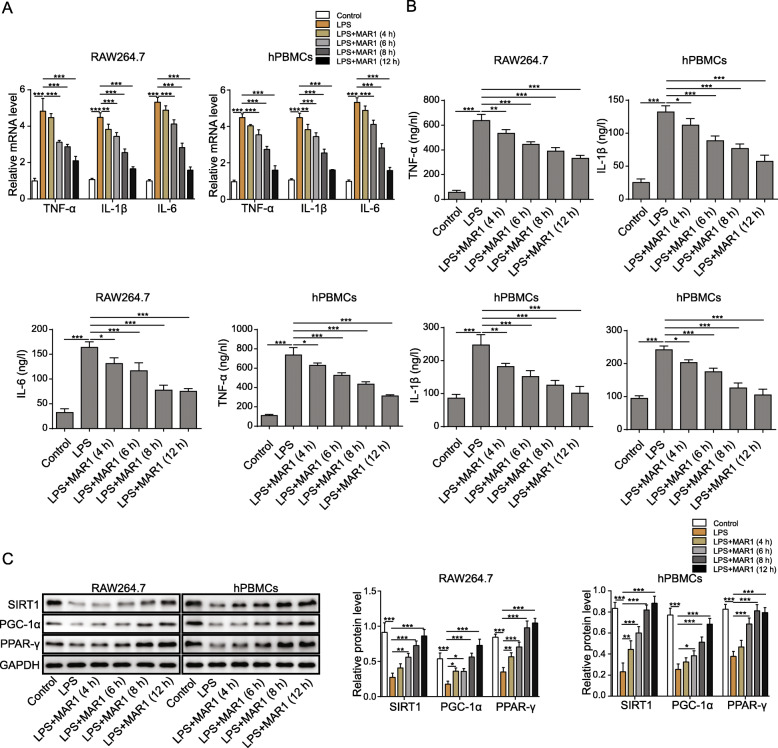
MAR1 inhibited the inflammatory response of RAW264.7 cells and hPBMCs induced by LPS in a time-dependent manner. RAW264.7 cells and hPBMCs were pretreated with MAR1 (10 nM) and then exposed to LPS (10 µg/mL) for 4, 6, 8, 12 h. **a**, TNF-α, IL-1β and IL-6 expression were detected by RT-qPCR; **b**, TNF-α, IL-1β and IL-6 secretion were measured using ELISA. **c**, The protein levels of SIRT1, PGC-1α and PPAR-γwere detected by Western blot. Data were shown as the means ± SD. Each experiment was repeated in triplicate. *p < 0.05, **p < 0.01, ***p < 0.001

The above results indicate that MAR1 reduces the inflammatory response of both RAW264.7 cells and hPBMCs induced by LPS in a dose-dependent manner especially at the highest concentration of 100 nM. We then consider investigated cells treated by 10 nM MAR1 by different time duration (0 h, 4 h, 6 h, 8 h and 12 h). The changes of inflammatory factors in LPS-treated RAW264.7 cells and hPBMCs at different intervention time (0 h, 4 h, 6 h, 8 h and 12 h) of MAR1 were analyzed by RT-qPCR. And we demonstrated that MAR1 inhibited the LPS-induced increase of the mRNA levels of proinflammatory factors in both RAW264.7 cells and hPBMCs with the treatment time (Fig. 2a). Then, we tested the secretion of TNF-α, IL-1β, and IL-6 in LPS-induced RAW264.7 cells or hPBMCs and the effect of MAR1 on their secretion. The results showed that LPS increased the secretion of TNF-α, IL-6 and IL-1β in both RAW264.7 cells and hPBMCs, which can be reversed by MAR1 (10 nM) (Fig. 2b). Thus, MAR1 reduced the levels of inflammatory factors with the treatment time. Additionally, we also performed the Western blot to evaluate the levels of SIRT1, PGC-1α and PPAR-γ protein, and the results showed that the expression of SIRT1, PGC-1α and PPAR-γ were significantly down-regulated in both RAW264.7 cells and hPBMCs induced by LPS (*p* < 0.001) (Fig. 2c) and were upregulated by MAR1 (10 nM) in a time-dependent manner. These results indicate that MAR1 reduces the inflammatory response induced by LPS in a time-dependent manner.

### PPAR-γ knockdown suppressed the effect of MAR1 on LPS-induced inflammatory response in RAW264.7 cells and hPBMCs

**Fig. 3:**
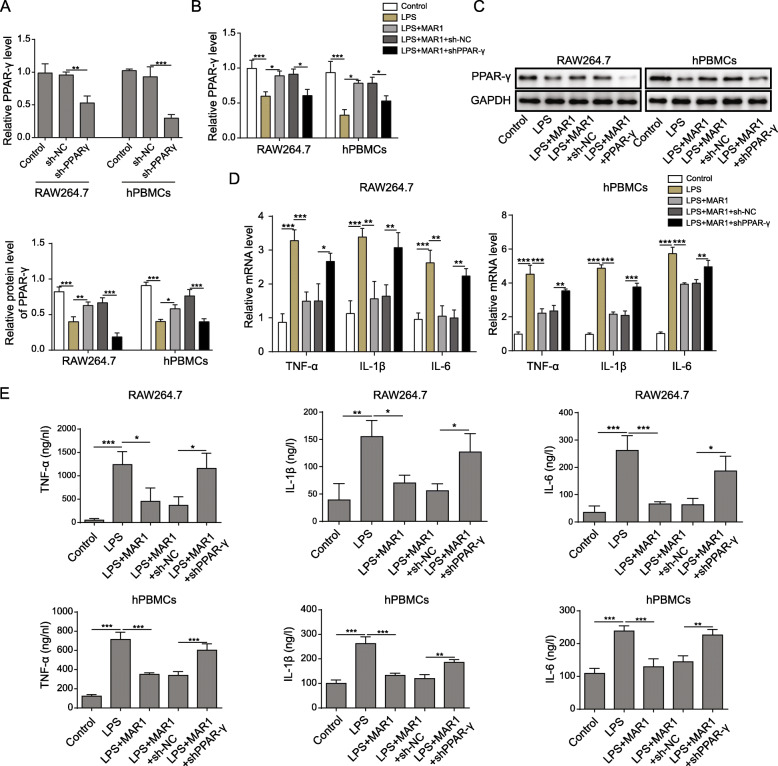
PPARγ knockdown suppressed the effect of MAR1 on LPS-induced inflammatory response in RAW264.7 cells and hPBMCs. Cells were treated with LPS (10 µg/ mL) for 24 h or treated with 10 nM of MAR1 for 12 h before LPS treatment. Each experiment was repeated in triplicate. **a**, Efficiency of RNA interfering was assayed by RT-qPCR. **b**, The protein level of PPAR-γ was detected by Western blot in different cell groups; **c**, The mRNA levels of TNF-α, IL-1β and IL-6 were determined by RT-qPCR in different cell groups. **d**, TNF-α, IL-1β and IL-6 secretion were measured using ELISA. Data were shown as the means ± SD. *p < 0.05, **p < 0.01, ***p < 0.001

Previous results revealed a possible mechanism for MAR1 in LPS-induced sepsis and inhibited inflammatory response in RAW264.7 cells or hPBMCs. In order to verify the role of PPAR-γ in the effect of MAR1 on LPS-induced sepsis, sepsis model of RAW264.7 cells and hPBMCs were induced by LPS and MAR1 (10 nM) for 12 h after sh-PPAR-γ and control vectors were stalely transfected. The results of RT-qPCR showed that the mRNA expression of PPAR-γ was markedly suppressed following transfection with the shRNA-containing vector (Fig. 3a). Both mRNA and protein levels of PPAR-γ significantly were decreased by LPS treatment, and this effect could be blocked by MAR1 treatment, which was further reversed by PPAR-γ knockdown (Fig. 3b and c). Both mRNA expression and protein levels of pro-inflammatory factors were suppressed under the action of MAR1 in LPS-induced RAW264.7 cells and hPBMCs, and the changes were reversed in cells with knockdown of PPAR-γ (Fig. 3d and e). Therefore, inhibition of PPAR-γ can offset the effect of MAR1 on inflammatory response in LPS-induced sepsis model.

### MAR1 suppressed inflammatory response in LPS-induced RAW 264.7 cells and hPBMCs via the SIRT1/PGC-1α/PPAR-γ pathway

**Fig. 4:**
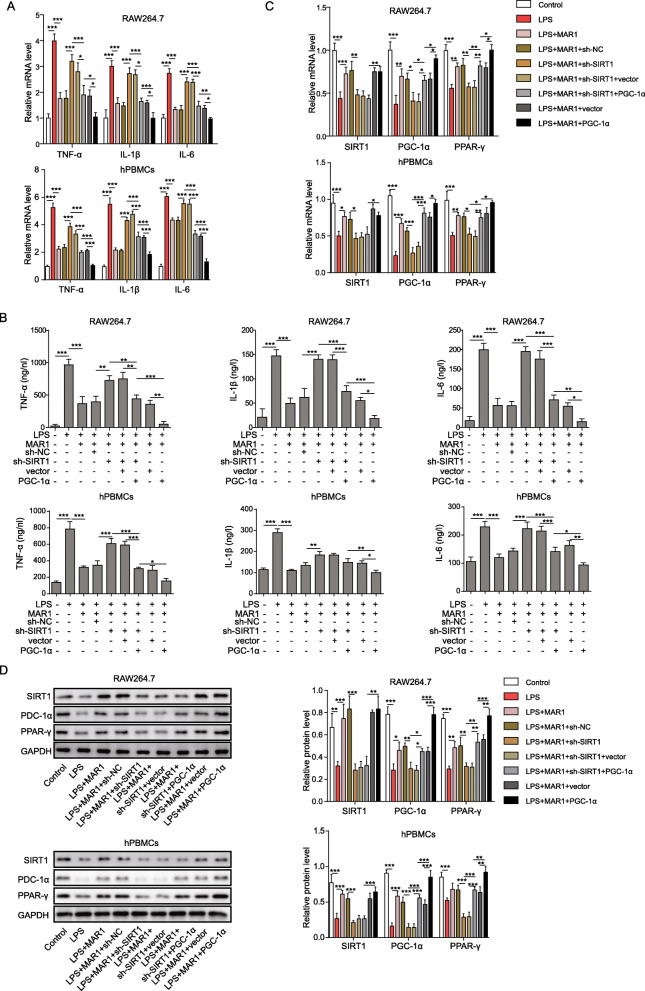
MAR1 suppressed inflammatory response in LPS-induced RAW 264.7 cells and hPBMCs via the SIRT1/PGC-1α/PPAR-γ pathway. Cells were treated with LPS (10 µg/ mL) for 24 h or treated with 10 nM of MAR1 for 12 h before LPS treatment. **a**, TNF-α, IL-1β and IL-6 expression were detected by RT-qPCR; **b**, TNF-α, IL-1β and IL-6 secretion were measured using ELISA. **c-d**, The mRNA and protein levels of SIRT1, PGC-1α and PPAR-γ were detected by RT-qPCR and Western blot, respectively. Data were shown as the means ± SD. Each experiment was repeated in triplicate. *p < 0.05, **p < 0.01, ***p < 0.001

The expression of TNF-α, IL-1β, and IL-6 induced by LPS were significantly reduced after treatment with MAR1 (*P* < 0.001), the effect of MAR1 can be counteracted by SIRT1 knockdown and further enhanced by PGC-1α overexpression (Fig. 4a). The changes in secretion of pro-inflammatory factors were examined by ELISA experiments. Secretion of TNF-α, IL-1β and IL-6 induced by LPS were significantly reduced after treatment with MAR1 (*P* < 0.001), the effect of MAR1 can be abolished by SIRT1 knockdown and further augmented by PGC-1α overexpression (Fig. 4b). Additionally, the mRNA and protein results in both RAW264.7 cells and hPBMCs showed that the expression of SIRT1, PGC-1α and PPAR-γ in the LPS group were significantly lower than those in the control group (*P* < 0.001), while MAR1 could restore their expression, and the effect of MAR1 can be counteracted by SIRT1 knockdown and further enhanced by PGC-1α overexpression (Fig. 4 c and d). These results indicate that MAR1 suppressed inflammatory response on LPS-induced sepsis via the SIRT1/PGC-1α/PPAR-γ pathway.

## Discussion

More and more studies have proved that using LPS-stimulated cells to establish an *in vitro* inflammation model can more closely study sepsis and LPS-treated macrophages were adopted as an *in vitro* model of endotoxin-induced inflammation during sepsis [9, 29, 30]. Recently, some studies have shown that MAR1 has a protective effect against inflammatory response by affecting SIRT1-mediated signaling pathway [31]. However, its underlying mechanism remains to be elucidated. In this study, we found that MAR1 attenuated inflammatory response by regulating the expression and secretion of LPS-induced proinflammatory cytokines. In addition, we also demonstrated that MAR1 increased SIRT1, PGC-1α, and PPAR-γ expression in RAW264.7 cells and hPBMCs. Then the cells were transfected with sh-SIRT1 or sh- PPAR-γ vector and PGC-1α overexpression vector to study the mechanism by which MAR1 abolish LPS-induced cellular inflammation. Therefore, this study aimed to determine the effect of MAR1 on inflammatory response and the relationship between MAR1 and SIRT1/PGC-1α/PPAR-γ axis in RAW264.7 cells and hPBMCs induced by LPS.

Sepsis is considered a serious disease with multiple organ damage caused by uncontrollable inflammation in severe infections, while MAR1 plays a role in reducing the bacterial burden and mitigating excessive inflammatory response and increases the survival rate of sepsis mice [32]. The study by Li et al. also suggests that the protective effect of MAR1 on sepsis may partly be related to the inhibition of the activation of NF-κB that causes increased proinflammatory cytokines such as IL-6, IL-1β and TNF-α in a mice model [33]. In addition, MAR1, as one of the newly discovered anti-inflammatory and pro-degradation mediators, can inhibit the infiltration and adhesion of neutrophils, enhance the macrophage phagocytosis of necrotic cells, down-regulate the production of inflammatory mediators and induce the GSK3β anti-inflammatory axis in human monocytes to limit the excessive development of inflammation and promote the timely resolution of inflammation[34, 35]. Our study shows for the first time that MAR1 can reduce the expression and secretion of pro-inflammatory factors and has shown protection against LPS-induced RAW264.7 cell and hPMBCs sepsis model.

**Fig. 5:**
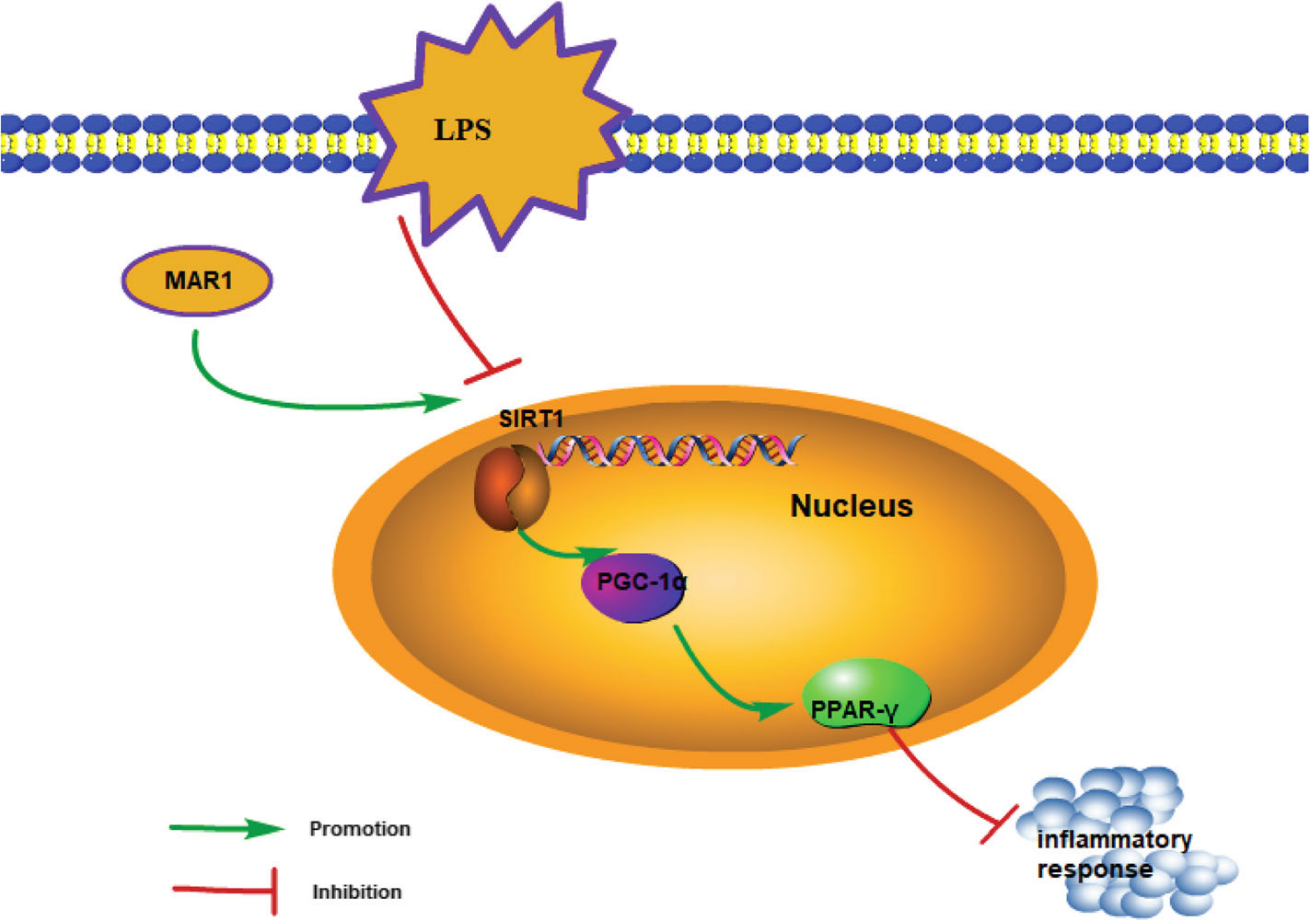
The schematic diagram consisting of identified pathway showing that MAR1 reduces the inflammatory response by activating the regulatory mechanism of the SIRT1/PGC-1α/PPAR-γ axis

There is evidence that increased expression of SIRT1 can reduce multiple organ damage, including lung, kidney, and liver, caused by sepsis [36, 37]. Interestingly, by activating SIRT1, MAR1 treatment exerted a neuroprotective effect in mice and thus had a therapeutic effect on cerebral ischemia-reperfusion injury [15]. Therefore, the protective effect of MAR1 on LPS-induced cellular inflammation in this study suggests that MAR1 may reduce the inflammatory response by activating SIRT1. In addition, the anti-inflammatory effects of SIRT1 in preventing multiple inflammatory response can be attributed to the activation of PGC-1α and the down-regulation of NF-κB [38]. PPAR-γ is a ligand-activated transcription factor involved in cell proliferation, lipid metabolism and inflammation. It has been shown to be a regulator of multiple inflammatory responses and activated PPAR-γ can improve survival in sepsis animals [27, 39]. Obviously, SIRT1 can reduce the production of various proinflammatory cytokines by activating PGC-1α and inhibiting NF-κB activation to reduce the acute inflammatory response in tissue injury [40]. In this study, we demonstrated that the levels of SIRT1, PGC-1α, and PPAR-γ protein involved in anti-inflammatory action were reduced in RAW264.7 cells and hPBMCs induced by LPS, while the proinflammatory cytokines IL-6, IL-1 β and TNF-α expression and secretion increased. Under the action of MAR1, the elevated levels of SIRT1, PGC-1α, and PPAR-γ protein and inhibited the expression of pro-inflammatory factors and exerted anti-inflammatory effects were observed. In subsequent observations, we inhibited the expression of SIRT1 in RAW264.7 cells and hPBMCs by transfected with shRNA against SIRT1 to explore the role of SIRT1 in the anti-inflammatory activity of MAR1. The results showed that the expression of PGC-1α and PPAR-γ protein was inhibited, the content of pro-inflammatory factors increased, and LPS-induced sepsis worsened. It was proved that SIRT1 was activated in RAW264.7 cells and hPBMCs under MAR1 treatment to reduce LPS-induced inflammatory response. At the same time, effect of suppressing SIRT1 expression was reversed by overexpression of PGC-1α. In summary, it can be concluded from the results of this study that MAR1 reduces the inflammatory response by activating the regulatory mechanism of the SIRT1/PGC-1α/ PPAR-γ axis (Fig. 5).

The present study also has some limitations. It is an *in vitro* study, thus the *in vivo* effects of MAR1 on sepsis as well as its clinical effects are not clear. More researches are still necessary to confirm the *in vivo* and potential clinical application of MAR1 in sepsis treatment.

In conclusion, this study demonstrates that the SIRT1/PGC-1α/PPAR-γ axis plays an important role in the process of MAR1 in reducing LPS-induced inflammation in *in vitro* sepsis model and provides clues for further exploring the mechanism of MAR1 in reducing inflammation in sepsis. Altogether, we propose that MAR1 could not only be a potential biomarker but also a novel therapeutic target for sepsis.

## Data Availability

All data generated or analysed during this study are included in this published article [and its supplementary information files].
